# The concept of natural genome reconstruction.Part 1. Basic provisions of the “natural genome reconstruction” concept. Changing the genome of hematopoietic stem cells using several natural cellular mechanisms that are inherent in the hematopoietic cell and determine its biological status as “the source of the body’s reparative potential”

**DOI:** 10.18699/vjgb-24-78

**Published:** 2024-11

**Authors:** L.A. Yakubov, O.S. Taranov, S.V. Sidorov, S.D. Nikonov, A.A. Ostanin, E.R. Chernykh, N.A. Kolchanov, S.S. Bogachev

**Affiliations:** State Scientific Center of Virology and Biotechnology “Vector” of Rospotrebnadzor, Koltsovo, Novosibirsk region, Russia; City Clinical Hospital No. 1, Novosibirsk, Russia; Novosibirsk Tuberculosis Research Institute, Novosibirsk, Russia; Research Institute of Fundamental and Clinical Immunology, Novosibirsk, Russia; Research Institute of Fundamental and Clinical Immunology, Novosibirsk, Russia; Institute of Cytology and Genetics of the Siberian Branch of the Russian Academy of Sciences, Novosibirsk, Russia; Institute of Cytology and Genetics of the Siberian Branch of the Russian Academy of Sciences, Novosibirsk, Russia

**Keywords:** extracellular DNA, internalization, single-strand breaks, commitment, экстраклеточная ДНК, интернализация, одноцепочечные разрывы, коммитирование

## Abstract

We present a series of articles proving the existence of a previously unknown mechanism of interaction between hematopoietic stem cells and extracellular double-stranded DNA (and, in particular, double-stranded DNA of the peripheral bloodstream), which explains the possibility of emergence and fixation of genetic information contained in double-stranded DNA of extracellular origin in hematopoietic stem cells. The concept of the possibility of stochastic or targeted changes in the genome of hematopoietic stem cells is formulated based on the discovery of new, previously unknown biological properties of poorly differentiated hematopoietic precursors. The main provisions of the concept are as follows. The hematopoietic stem cell takes up and internalizes fragments of extracellular double-stranded DNA via a natural mechanism. Specific groups of glycocalyx factors, including glycoproteins/proteoglycans, glycosylphosphatidylinositol-anchored proteins and scavenger receptors, take part in the internalization event. The binding sites for DNA fragments are heparin-binding domains and clusters of positively charged amino acid residues that are parts of protein molecules of these factors. Extracellular fragments delivered to the internal compartments of hematopoietic stem cells initiate terminal differentiation, colony formation, and proliferation of hematopoietic precursors. The molecular manifestation of these processes is the emergence and repair of pangenomic single-strand breaks. The occurrence of pangenomic single-strand breaks and restoration of genome (genomic DNA) integrity are associated with activation of a “recombinogenic situation” in the cell; during its active phase, stochastic homologous recombination or other recombination events between extracellular fragments localized in the nucleus and chromosomal DNA are possible. As a result, genetic material of initially extracellular localization either integrates into the recipient genome with the replacement of homologous chromosomal segments, or is transitively present in the nucleus and can manifest itself as a new genetic trait. It is assumed that as a result of stochastic acts of homologous exchange, chromosome loci are corrected in hematopoietic stem cells that have acquired mutations during the existence of the organism, which are the cause of clonal hematopoiesis associated with old age. In this regard, there is a fundamental possibility of changing the hematopoietic status of hematopoietic stem cells in the direction of polyclonality and the original diversity of clones. Such events can form the basis for the rejuvenation of the blood-forming cell system. The results of the laboratory’s work indicate that other stem cells in the body capture extracellular DNA fragments too. This fact creates a paradigm for the overall rejuvenation of the body.

## Abstract (ru)

Предлагается вниманию цикл статей, доказывающий существование ранее неизвестного механизма взаимодействия гемопоэтической стволовой клетки и экстраклеточной двуцепочечной ДНК (в частности, двуцепочечной ДНК периферического кровяного русла), который объясняет возможность появления и закрепления в гемопоэтических стволовых клетках генетической информации, содержащейся в двуцепочечной ДНК внеклеточного происхождения. Сформулирована концепция возможности стохастического или целенаправленного изменения генома гемопоэтических стволовых клеток, основанная на открытии новых, ранее неизвестных биологических свойств низкодифференцированных гемопоэтических предшественников. Основные положения концепции заключаются в следующих тезисах. Гемопоэтическая стволовая клетка захватывает и интернализует фрагменты экстраклеточной двуцепочечной ДНК естественным природным механизмом. В акте интернализации принимают участие специфические группы факторов гликокаликса, к которым относятся гликопротеины/ протеогликаны, гликозилфосфатидилинозитол-заякоренные белки и скавенджер-рецепторы. Сайтами связывания фрагментов ДНК являются гепарин-связывающие домены и кластеры положительно заряженных аминокислотных остатков, входящих в состав белковых молекул указанных факторов. Доставленные во внутренние компартменты гемопоэтических стволовых клеток экстраклеточные фрагменты инициируют терминальную дифференцировку, колониеобразование и пролиферацию предшественников гемопоэза. Молекулярным событием, отражающим эти процессы, является возникновение и репарация пангеномных одноцепочечных разрывов. Процесс возникновения пангеномных одноцепочечных разрывов и восстановление целостности генома (геномной ДНК) сопряжен с активацией в клетке «рекомбиногенной ситуации», во время активной фазы которой возможны стохастическая гомологичная рекомбинация или иные рекомбинационные события между экстраклеточными фрагментами, локализованными в ядре, и ДНК хромосом. Генетический материал исходно экстраклеточной локализации или интегрирует в реципиентный геном с замещением гомологичных хромосомных сегментов, или транзитно присутствует в ядре и может проявляться как новый генетический признак. Предпола гается, что в результате стохастических актов гомологичного обмена происходит коррекция локусов хромосом в гемопоэтических стволовых клетках, получивших в ходе существования организма мутации, которые являются причиной клонального гемопоэза, ассоциированного со старостью. В этой связи возникает принципиальная возможность изменения статуса гемопоэза гемопоэтических стволовых клеток в направлении поликлонально сти и исходного многообразия клонов. Такие события могут составить основу омоложения кровеобразующей системы клеток. Результаты работ свидетельствуют, что другие стволовые клетки организма также захватывают фрагменты экстраклеточной ДНК. Этот факт создает парадигму общего омоложения организма.

## Double-stranded DNA and its effects on a eukaryotic cell and the organism in general

Double-stranded fragmented extracellular exogenous and endogenous DNA is a participant, inducer and indicator of various processes occurring in the body. First and foremost, exogenous nucleic acids are pathogen-associated molecular patterns that activate different components of the immune system aimed at pathogen removal. Extracellular double-stranded DNA (dsDNA) of endogenous origin can also elicit the body’s immune response (Medzhitov, Janeway, 2000; Krieg, 2002; Takeshita, Ishii, 2008; Iwasaki, Medzhitov, 2010; Barbalat et al., 2011; Bode et al., 2011). Fragments of both exogenous and endogenous dsDNA delivered to the cytoplasm of immune cells activate an array of cytosolic DNA sensors and induce the early stages of adaptive immune response (Barber, 2011a, b; Sharma, Fitzgerald, 2011). dsDNA induces autoimmune processes Guiducci et al., 2010; Pisetsky, Ullal, 2010; Almqvist et al., 2011; Choubey, 2012; Kaczorowski et al., 2012) and is one of the signals of the bystander effect responsible for transmission of metabolic/catabolic reactions induced in cells exposed to a certain impact (irradiation) to intact bystander cells through the incubation medium (Ermakov et al., 2009). The burst-like increase in plasma DNA concentration has been shown to result in systemic inflammatory response and sepsis (Saukkonen et al., 2007; Castellheim et al., 2009; Arnalich et al., 2010; Kaczorowski et al., 2012). Nucleic acids, including dsDNA, are components of exosomes and are believed to act as a “tuning fork” for the functional state of the organism used by certain cell populations to “fine-tune” physiological and molecular processes in them (Cai et al., 2013; Rashed et al., 2017).

At the cellular level, dsDNA fragments (namely, open double-strand ends of these fragments) internalized by a non-immune cell activate cell cycle arrest and induce repair processes (MacDougall et al., 2007; Zou, 2007). Meanwhile, these fragments become involved in repair under certain conditions, interfering in its correct course; according to the experimental laboratory data, they can be integrated into the recipient genome (Likhacheva et al., 2007, 2008; Dolgova et al., 2012).

Horizontal gene transfer has been studied well in prokaryotes and repeatedly demonstrated for lower metazoans (Andersson, 2005; Soucy et al., 2015; Sibbald et al., 2020); there are proven examples of gene transfer in mammals. Thus, it is known that DNA of destroyed cancer cells is taken up by other susceptible cells in the body, resulting in malignant transformation of these cells. This process is called “genometastasis” (Yakubov et al., 2007; García-Olmo et al., 2012). Evidence for horizontal gene transfer during the uptake of apoptotic bodies and extracellular vesicles has been provided in refs. (Bergsmedh et al., 2001; Holmgren et al., 2002; Sakamoto et al., 2023). There is a study focusing on transfer of extracellular material into an oocyte by spermatozoa that have engulfed high-polymeric DNA from the environment in mussels (Erokhin, Kuznetsov, 2009). Horizontal transfer of a specific unique gene between an insect and a plant has been demonstrated recently (Xia et al., 2021). In 2007, the Nobel Prize was awarded for developing a technology based on homologous recombination of internalized knockout DNA fragments (https://lenta.ru/articles/2007/10/08/nobelmed/).

Several fundamental questions have been consecutively solved as part of the problem of horizonal gene transfer in eukaryotes and humans in particular. The questions related to how the DNA of one organism appeared in another one and the mechanism of DNA spread across the organism have been solved using novel experimental approaches, modern molecular biology techniques. It still remains unclear what is the fixation point of new genetic information in the recipient organism and how a trait associated with it manifests itself as a new biological feature of a new organism

There are several objective sources and pathways through which foreign DNA appears in the organism: blood manipulations, various transplantations, sexual intercourse, gestation, passage of DNA from food to the organism, viral or bacterial infection, and shared microbiome of the organism.

After it had been proved that peripheral blood of any organism contains a certain quantity of extracellular DNA, the question related to existence of foreign DNA and its spread in the organism was also solved (Anker et al., 1999; Jahr et al., 2001; Laktionov et al., 2004). It is clear that during blood manipulations and transplantation, donor DNA (blood or stroma) necessarily gets into the recipient organism. DNA of damaged spermatozoa, like fetal DNA, gets into the blood and vice versa (Schubbert et al., 1998; Bianchi, Dennis Lo, 2001). Emergence of exogenous DNA through the gastrointestinal tract has been convincingly demonstrated in refs. (Schubbert et al., 1994, 1997; Hohlweg, Doerfler, 2001; Palka-Santini et al., 2003). The gut microbiota is composed of several kilograms of its main constituent species, Escherichia coli. Destruction of intestinal cells is accompanied by release of a massive amount of DNA that also gets into the bloodstream. This very phenomenon has underlain methods for diagnosing the state of the gut microflora by analyzing blood serum/ plasma that have been developed and launched into clinical practice (https://lenta.ru/articles/2007/10/08/nobelmed/). It means that along with endogenous DNA from cells destroyed by apoptosis or necrosis or DNA of symbiotic microflora, the organism will always contain exogenous DNA, and there are material foundations for the circulation of both foreign and endogenous genetic information in the organism.

Currently, there is almost no discussion on the final question and related ideas about the fixation point of new genetic information and its manifestation as a novel biological trait in scientific literature, since there are no ideas on how these questions can be solved.

It is clear that for a trait to manifest itself, its carrier (namely, dsDNA) needs to be internalized by the cell. There currently exist many publications demonstrating the fact that extracellular DNA fragments are internalized by different cell types; however, it was not until our studies had been published (Petrova et al., 2022; Ritter et al., 2022; Potter et al., 2024) that the mechanism and the ordered structure of factors of this internalization were described. The cited papers revealed that fragments of extracellular dsDNA are internalized by the eukaryotic stem cell via the caveolae-dependent mechanism, with involvement of the heparin-binding domain and clusters of positively charged amino acids of glycoproteins/proteoglycans, glycosylphosphatidylinositol-anchored proteins, and scavenger receptors of the glycocalyx. The total positive charge of the stem cell of different genesis is the key factor for internalization

A viewpoint is currently being formed assuming that extracellular nucleic acids, including dsDNA, refer to a new type of regulatory system of the organism having complex mechanisms of regulation of cellular process; horizontal gene transfer being one of its manifestations (Ledoux, 1965; Ratajczak et al., 2006; Cocucci et al., 2009; Simons, Raposo, 2009; Camussi et al., 2010; Balaj et al., 2011; Tetta et al., 2011; Ludwig, Giebel, 2012; Ronquist, 2012; Raposo, Stoorvogel, 2013).

## History of the development of the natural genome reconstruction concept

The concept of natural genome reconstruction – the ability to reconstruct pathologically altered chromatin topology (both changes at the nucleotide level and related higher-order changes in chromatin organization) – is the feasibility of introducing molecular changes (“corrections” in the case when it is the cause of a disease) in mutant chromosomal loci (or “healthy-to-healthy” replacement without altering the primary nucleotide sequence) in vivo, ex vivo, and in situ, which employs the principle of a supplementary reconstructive substrate as “genetic material” (fragments of extracellular dsDNA).

The concept is based on five fundamental phenomena of general biology; three of those have recently been discovered and described for the stem cell at the Laboratory of Induced Cellular Processes (Institute of Cytology and Genetics of the Siberian Branch of the Russian Academy of Sciences, Novosibirsk, Russia).

**Fig. 1.**
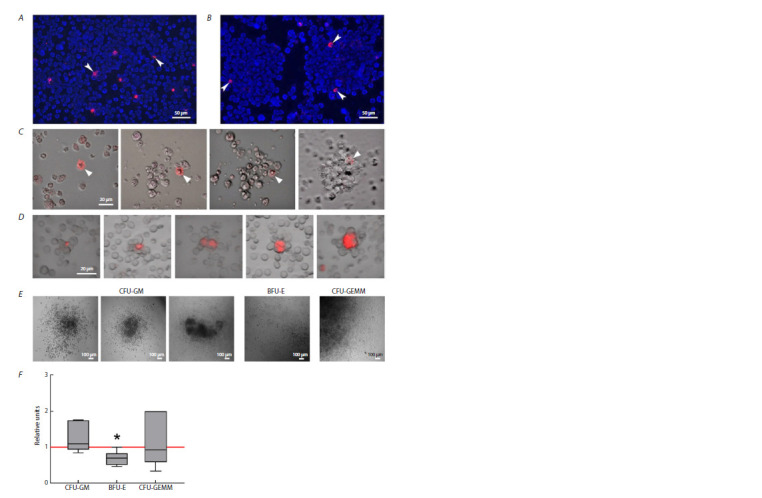
Internalization of TAMRA-labeled DNA probe into stem cells from Krebs-2 carcinoma (A) and Epstein–Barr virustransformed B-cell lymphoma (B). Arrows show TAMRA+ cells. One can see two spheres in Figure 1B. C – formation of spheres in the culture of Epstein–Barr virus-transformed lymphoma cells. A TAMRA+ cell merges with several adjacent cells within 20–30 min. During the observation, all the cells within the visual field are continuously moving chaotically. A strong associate is formed when cells contact the TAMRA+ cell; a free-floating sphere based on it is formed within 8–14 hrs (Dolgova et al., 2016, 2019). D – rosettes formed by bone marrow stromal niches, with TAMRA+ cells located in the center (Ritter et al., 2022). E – morphologies of the analyzed colonies of the granulocyte-macrophage (CFU-GM), erythroid (BFU-E), and myeloid (CFU-GEMM) hematopoietic lineage upon induction with hDNAgr. F – stimulation of colony formation of the granulocytemacrophage (CFU-GM), erythroid (BFU-E), and myeloid (CFU-GEMM) hematopoietic lineages upon induction with hDNAgr compared to control. The values are the ratio between the number of colonies upon induction with the preparation and the number of control colonies assumed to be equal to “1” and denoted with the red line. The data are presented as the median, the interquartile range, and the minimum and maximum values. * – statistically significant differences compared to the control group (p < 0.05, Wilcoxon matched pairs test, n = 6) (Potter et al., 2024).

**Fig. 2.**
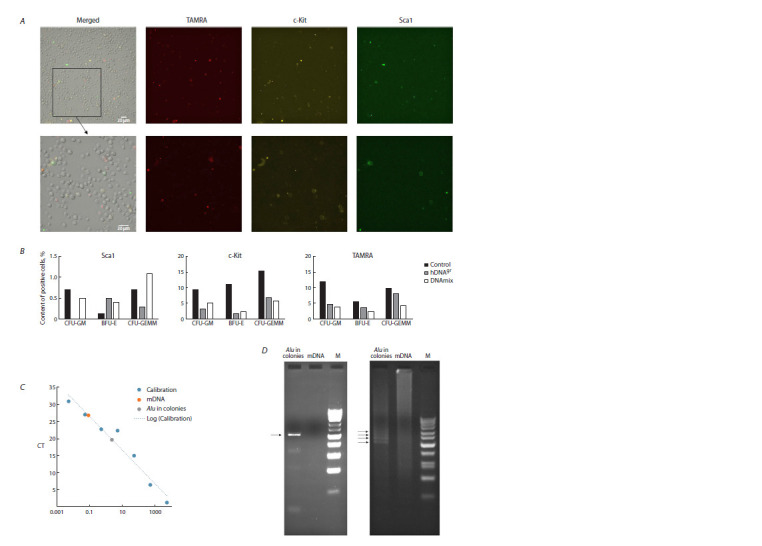
Analysis of internalization of double-stranded DNA probe in HSCs. A – assessment of colocalization of markers of primitive HSCs c-Kit and Sca1 and simultaneously the ability of these cells to internalize TAMRA+ double-stranded DNA probe in intact murine bone marrow. B – comparative diagrams of the contents of cells labeled with the respective marker in cell population of an individual colony. The reduced number of cells labelled with stemness markers may indicate that induction with DNA preparation caused stem cell differentiation. C – analysis of internalization of double-stranded DNA repeat probe AluI in HSCs Sca1/c-Kit by real-time PCR. Calculations indicate that the content of the DNA probe is ~14,000 copies per cell. D – electrophoretic mobility of the PCR fragment obtained using primers M13 and DNA template isolated from murine HSCs, induced by hDNAgr, and incubated in the presence of human Alu fragment. On the left: PCR products from templates “Alu in colonies” and murine DNA. On the right: electrophoresis of real-time PCR products from the same templates. Arrows show the PCR products synthesized from concatemerized DNA template AluI (Potter et al., 2024).

**Fig. 3.**
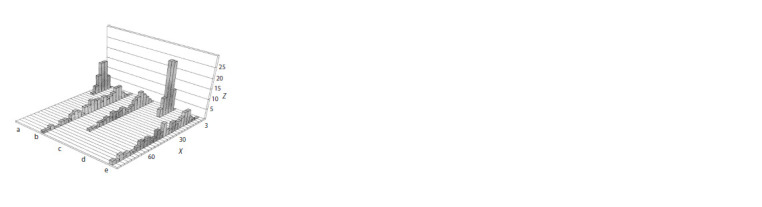
The histogram showing distribution of DNA migration path length of OTF9-63 cells before and after cultivation during different time periods in the presence of retinoic acid. X – DNA migration path length in μm (scale division value, 3 μm); Z – number of cells: a – undifferentiated cells; b–d – differentiation during 24, 36, and 96 hrs, respectively; e – undifferentiated embryonic stem cells irradiated with X-rays at a dose of 200 rad. Among cells exposed to radiation, migration path length involves the nucleus size (Vatolin et al., 1997).

1. Presence of fragments of extracellular dsDNA in peripheral blood (Anker et al., 1999; Jahr et al., 2001; Laktionov et al., 2004).

2. Ability of stem cells of different genesis to internalize fragments of double-stranded DNA via a natural mechanism (Fig. 1). The following factors are involved in the internalization mechanism: cell surface charge, heparin-binding domains and clusters of positively charged amino acids, glycoproteins/proteoglycans, and scavenger receptors of the glycocalyx. Internalization occurs via the caveolae/ clathrin-dependent pathway (Dolgova et al., 2014; Petrova et al., 2022; Ritter et al., 2022).

3. Induction of terminal differentiation of stem cells by ds- DNA fragments internalized by intracellular compartments (Fig. 2). Using the mouse model, we have found that extracellular dsDNA fragments represented by PCR fragments, supercoiled plasmid DNA, and fragmented genomic dsDNA are internalized by hematopoietic stem cells (HSCs) via a natural mechanism. Intracellular compartments of c-Kit+/ Sca1+/CD34+ HSCs and multipotent descendants can simultaneously contain up to 14,000 copies of ~500 bp long PCR fragments (~0.2 % of the haploid genome). Internalized dsDNA fragments induce terminal differentiation of the progenitor, activate proliferation of descendant cells and colony growth on microcrystalline cellulose. The number of colonies increases by 15–40 % compared to that for untreated samples (Potter et al., 2024). In colonies, most cells (for the mouse model) are terminally differentiated ones in which markers of primitive progenitor cells (c-Kit+/Sca1+ for mice) are lost. A smaller part of cells (up to 15 %) retain markers of immature cells. This fact indicated that upon colony formation, HSCs primarily activated by exposure to Lin–/c-Kit+/Sca1+ divide both asymmetrically (yielding committed hematopoietic progenitor cells) and symmetrically (increasing the pool of poorly differentiated progenitor cells) (Potter et al., 2024). The number of HSCs in the total pool of cells within colonies was several orders of magnitude larger than that in the original sample of bone marrow cells. This fact indicates that the mechanism responsible for amplification (up to 15 % of primitive cells for the mouse model) of a new trait that initially appeared in HSCs of bone marrow cells (0.01 % of primitive cells) has been found.

4. It was found previously that pangenomic single-strand breaks are formed in mammalian embryonic stem cells after induction of their terminal differentiation by treatment with retinoic acid; according to the published data, these breaks are restored within 96 hrs (Vatolin et al., 1997) (Fig. 3). The emergence of pangenomic single-strand breaks causes conversion of nuclear chromatin to its relaxed form. Further repair of single-strand breaks results in restoration of genome integrity and formation of a new 3D chromatin architecture taking into account new torsional strains that are responsible for its new 3D architecture in the selected differentiation direction. It is believed that this genomic “opening and relaxation” is needed to ensure chromatin to- pology reorganization from the “stem” state to the lineagecommitted one (Farzaneh et al., 1982; Johnstone, Williams, 1982). The emergence and repair of pangenomic singlestrand breaks is a purely recombination repair process indicating that a “recombinogenic situation” has occurred in the cell (Likhacheva et al., 2008). The molecular machinery of recombination repair involved in chromatin break and repair is unknown.

5. The ability of internalized dsDNA fragments to participate in repair processes (induced either by fragments per se or by other factors) occurring in the cell either as a substrate or as the external template for homologous recombination (Dolgova et al., 2014; Potter et al., 2016, 2017, 2024; Ruzanova et al., 2022).

The aforementioned announcement of experimental materials demonstrates that internalization of dsDNA by a HSC induces terminal differentiation (commitment) and activates proliferation progenitors associated with it. We believe that similar to terminal differentiation and emergence of pangenomic single-strand breaks observed in embryonic stem cells, which were demonstrated in ref. (Vatolin et al., 1997), pangenomic single-strand breaks are also induced in HSCs upon commitment, thus leading to the development of a “recombinogenic situation” (Likhacheva et al., 2008). At this very instant, extracellular DNA fragments internalized by stem cells become natural participants of recombinant processes, which are associated with the emergence and repair of these single-strand breaks.

A hypothesis has been put forward that during a “recombinogenic situation” developed in HSCs, different recombination repair events can occur between captured extracellular DNA fragments residing inside the cell, which have induced the emergence of single-strand breaks and the “recombinogenic situation”, and chromosomal DNA. These recombination repair events result in alteration of certain chromosomal loci within the repair zone mediated by natural molecular processes because of either homologous exchange or integration of accessory chromatin (e. g., into centromeric or telomeric agglomerates). Transient repair intermediates may appear. These events will lead to integration of extracellular fragments into the recipient genome, or reconstructive replacement of homologous genomic loci, or emergence of transient repair intermediates not integrated into the genome, with consequent biological changes at the cellular, tissue, organ, functional system, and organism levels

It is supposed that nonhomologous integration of extracellular fragments is hardly feasible because there are no molecular foundations for it (namely, nonfunctional double-strand breaks). The feasibility of fixation of nonhomologous extracellular DNA in the genome can be related to the imperfection of the mechanism of single-strand annealing and involvement of microhomologies in this process

## The concept of natural genome reconstruction

Extracellular dsDNA fragments are taken up and internalized into compartments of HSCs via a natural mechanism. The aforementioned substrate (extracellular dsDNA fragments) is “offered” to a stem cell as extracellular material or under in vivo, ex vivo, or in situ conditions. dsDNA fragments internalized into cellular compartments induce terminal differentiation of hematopoietic stem cells; emergence of pangenomic single-strand breaks that extracellular fragments located inside cells interact with is one of its markers. The pangenomic single-strand breaks ensure the relaxed form of a certain level of nuclear chromatin compaction, thus providing conditions for subsequent chromatin reorganization. Chromatin in this state undergoes mitosis. The first mitotic division results in restoration of the 3D chromatin architecture of the original cell with stem characteristics in one daughter cell. In the second daughter cell, chromatin reorganization (“reprogramming”) takes place, and a new 3D architecture in the selected direction of cell differentiation is formed with allowance for a new torsional strain responsible for the new 3D structure of chromatin. A “recombinogenic situation” is induced in the HSC at the instant when pangenomic singlestrand breaks appear and are repaired, and conditions for integrating fragments internalized by the cell into chromatin DNA are established. Either replacement of homologous regions or direct integration of extracellular fragments into homologous or non-homologous genomic regions occurs, or transient intermediates are formed. The choice of the pathway and the integration mechanism are unknown; however, homologous exchange between the extracellular template and genomic DNA is primarily suspected. HSCs in which “illegitimate” integration of extracellular nonhomologous DNA has taken place are eliminated from the HSC population. The mechanistic schematics of the aforementioned processes are provided in the thematic experimental sections of this series of studies

## Conclusion

The proposed concept has been proved in a number of experimental studies united together into a series of studies collectively called “The Concept of Natural Genome Reconstruction”. Further articles within this series will focus on questions characterizing early events of interaction between HSCs and extracellular fragments of dsDNA, as well as the implications of this interaction altering both the structural and linear arrangement of extracellular dsDNA fragments, as well as the functional state of target hematopoietic progenitor cells. Genomic changes related to the emergence of extracellular dsDNA fragments in the intracellular space of stem cells will be analyzed. In vivo tests will assess the effect of changes in the genome of HSCs caused by interaction with extracellular dsDNA fragments on certain biological parameters of experimental animals (changes in lifespan). Finally, the pleiotropic effect of the impact of extracellular dsDNA on human HSCs will be analyzed in a clinical case study, with special emphasis placed on the shift in the hematopoietic status (oligoclonal/ normal).

## Conflict of interest

The authors declare no conflict of interest.

## References

Almqvist N., Winkler T.H., Mårtensson I.L. Autoantibodies: focus
on anti-DNA antibodies. Self/Nonself. 2011;2(1):11-18. DOI
10.4161/SELF.2.1.15087

Andersson J.O. Lateral gene transfer in eukaryotes. Cell. Mol.
Life Sci. 2005;62(11):1182-1197. DOI 10.1007/S00018-005-
4539-Z

Anker P., Mulcahy H., Chen X.Q., Stroun M. Detection of circulating
tumour DNA in the blood (plasma/serum) of cancer patients.
Cancer Metastasis Rev. 1999;18(1):65-73. DOI 10.1023/a:
1006260 319913

Arnalich F., Maldifassi M.C., Ciria E., Quesada A., Codoceo R.,
Herruzo
R., Garcia-Cerrada C., Montoya F., Vazquez J.J., López-
Collazo
E., Montiel C. Association of cell-free plasma DNA with
perioperative mortality in patients with suspected acute mesenteric
ischemia. Clin. Chim. Acta. 2010;411(17-18):1269-1274.
DOI 10.1016/j.cca.2010.05.017

Balaj L., Lessard R., Dai L., Cho Y.J., Pomeroy S.L., Breakefield
X.O., Skog J. Tumour microvesicles contain retrotransposon
elements and amplified oncogene sequences. Nat. Commun.
2011;2:180. DOI 10.1038/ncomms1180

Barbalat R., Ewald S.E., Mouchess M.L., Barton G.M. Nucleic acid
recognition
by the innate immune system. Annu. Rev. Immunol.
2011;29(1):185-214. DOI 10.1146/annurev-immunol-031210-
101340

Barber G.N. Cytoplasmic DNA innate immune pathways. Immunol.
Rev. 2011a;243(1):99-108. DOI 10.1111/j.1600-065X.2011.
01051.x

Barber G.N. Innate immune DNA sensing pathways: STING,
AIMII and the regulation of interferon production and inflammatory
responses. Curr. Opin. Immunol. 2011b;23(1):10-20. DOI
10.1016/ j.coi.2010.12.015

Bergsmedh A., Szeles A., Henriksson M., Bratt A., Folkman M.J.,
Spetz A.L., Holmgren L. Horizontal transfer of oncogenes by
uptake of apoptotic bodies. Proc. Natl. Acad. Sci. USA. 2001;
98(11):6407-6411. DOI 10.1073/PNAS.101129998

Bianchi D.W., Dennis Lo Y.M. Fetomaternal cellular and plasma
DNA trafficking: the Yin and the Yang. Ann. NY Acad. Sci. 2001;
945:119-131. DOI 10.1111/J.1749-6632.2001.TB03872.X

Bode C., Zhao G., Steinhagen F., Kinjo T., Klinman D.M. CpG
DNA as a vaccine adjuvant. Expert Rev. Vaccines. 2011;10(4):
499-511. DOI 10.1586/erv.10.174

Cai J., Han Y., Ren H., Chen C., He D., Zhou L., Eisner G.M.,
Asico
L.D., Jose P.A., Zeng C. Extracellular vesicle-mediated
transfer of donor genomic DNA to recipient cells is a novel
mechanism for genetic influence between cells. J. Mol. Cell Biol.
2013;5(4):227-238. DOI 10.1093/JMCB/MJT011

Camussi G., Deregibus M.C., Bruno S., Cantaluppi V., Biancone L.
Exosomes/microvesicles as a mechanism of cell-to-cell communication.
Kidney Int. 2010;78(9):838-848. DOI 10.1038/
KI.2010.278

Castellheim A., Brekke O.L., Espevik T., Harboe M., Mollnes T.E.
Innate
immune responses to danger signals in systemic inflammatory
response syndrome and sepsis. Scand. J. Immunol. 2009;
69(6):479-491. DOI 10.1111/j.1365-3083.2009.02255.x

Choubey D. DNA-responsive inflammasomes and their regulators
in autoimmunity. Clin. Immunol. 2012;142(3):223-231. DOI
10.1016/ j.clim.2011.12.007

Cocucci E., Racchetti G., Meldolesi J. Shedding microvesicles:
artefacts no more. Trends Cell Biol. 2009;19(2):43-51. DOI
10.1016/ J.TCB.2008.11.003

Dolgova E.V., Proskurina A.S., Nikolin V.P., Popova N.A., Alyamkina
E.A., Orishchenko K.E., Rogachev V.A., Efremov Y.R., Dubatolova
T.D., Prokopenko A.V., Chernykh E.R., Ostanin A.A.,
Taranov O.S., Omigov V.V., Zagrebelniy S.N., Bogachev S.S.,
Shurdov M.A. “Delayed death” phenomenon: a synergistic action
of cyclophosphamide and exogenous DNA. Gene. 2012;
495(2):134-145. DOI 10.1016/j.gene.2011.12.032

Dolgova E.V., Alyamkina E.A., Efremov Y.R., Nikolin V.P., Popova
N.A., Tyrinova T.V., Kozel A.V., Minkevich A.M., Andrushkevich
O.M., Zavyalov E.L., Romaschenko A.V., Bayborodin
S.I., Taranov O.S., Omigov V.V., Shevela E.Y., Stupak V.V.,
Mishinov S.V., Rogachev V.A., Proskurina A.S., Mayorov V.I.,
Shurdov M.A., Ostanin A.A., Chernykh E.R., Bogachev S.S.
Identification of cancer stem cells and a strategy for their
elimination. Cancer Biol. Ther. 2014;15(10):1378-1394. DOI
10.4161/cbt.29854

Dolgova E.V., Shevela E.Y., Tyrinova T.V., Minkevich A.M.,
Proskurina A.S., Potter E.A., Orishchenko K.E., Zavjalov E.L.,
Bayborodin S.I., Nikolin V.P., Popova N.A., Pronkina N.V., Ostanin
A.A., Chernykh E.R., Bogachev S.S. Nonadherent spheres
with multiple myeloma surface markers contain cells that contribute
to sphere formation
and are capable of internalizing extracellular
double-stranded DNA. Clin. Lymphoma Myeloma
Leuk. 2016;16(10):563-576. DOI 10.1016/j.clml.2016.06.014

Dolgova E.V., Petrova D.D., Proskurina A.S., Ritter G.S., Kisaretova
P.E., Potter E.A., Efremov Y.R., Bayborodin S.I., Karamysheva
T.V., Romanenko M.V., Netesov S.V., Taranov O.S.,
Ostanin A.A., Chernykh E.R., Bogachev S.S. Identification of
the xenograft and its ascendant sphere-forming cell line as belonging
to EBV-induced lymphoma, and characterization of the
status of sphere-forming cells. Cancer Cell Int. 2019;19(1):120.
DOI 10.1186/s12935-019-0842-x

Erokhin V.E., Kuznetzov A.V. Informative and regulatory roles of
dissolved organic matter in seawater. Morskoi Ekologicheskiy
Zhurnal = Marine Ecol. J. 2009;8(2):64-69 (in Russian)

Ermakov A.V., Konkova M.S., Kostyuk S.V., Egolina N.A., Efremova
L.V., Veiko N.N. Oxidative stress as a significant factor for
development of an adaptive response in irradiated and nonirradiated
human lymphocytes after inducing the bystander effect by
low-dose X-radiation. Mutat. Res. 2009;669(1-2):155-161. DOI
10.1016/ j.mrfmmm.2009.06.005

Farzaneh F., Zalin R., Brill D., Shall S. DNA strand breaks and
ADP-ribosyl transferase activation during cell differentiation.
Nature. 1982; 300(5890):362-366. DOI 10.1038/300362A0

García-Olmo D.C., Picazo M.G., García-Olmo D. Transformation
of non-tumor host cells during tumor progression: theories and
evidence. Expert Opin. Biol. Ther. 2012;12(Suppl.1):S199-207.
DOI 10.1517/14712598.2012.681370

Guiducci C., Gong M., Xu Z., Gill M., Chaussabel D., Meeker T.,
Chan J.H., Wright T., Punaro M., Bolland S., Soumelis V.,
Banchereau
J., Coffman R.L., Pascual V., Barrat F.J. TLR recognition
of self nucleic acids hampers glucocorticoid activity in
lupus. Nature. 2010;465(7300):937-941. DOI 10.1038/nature
09102

Hohlweg U., Doerfler W. On the fate of plant or other foreign genes
upon the uptake in food or after intramuscular injection in mice.
Mol. Genet. Genomics. 2001;265(2):225-233. DOI 10.1007/
S004380100450

Holmgren L., Bergsmedh A., Spetz A.L. Horizontal transfer of DNA
by the uptake of apoptotic bodies. Vox Sang. 2002;83(Suppl.1):
305-306. DOI 10.1111/J.1423-0410.2002.TB05323.X

Iwasaki A., Medzhitov R. Regulation of adaptive immunity by the
innate immune system. Science. 2010;327(5963):291-295. DOI
10.1126/science.1183021

Jahr S., Hentze H., Englisch S., Hardt D., Fackelmayer F.O.,
Hesch R.D., Knippers R. DNA fragments in the blood plasma of
cancer patients: quantitations and evidence for their origin from
apoptotic and necrotic cells. Cancer Res. 2001;61(4):1659-1665.
http://www.ncbi.nlm.nih.gov/pubmed/11245480

Johnstone A.P., Williams G.T. Role of DNA breaks and ADP-ribosyl
transferase activity in eukaryotic differentiation demonstrated
in human lymphocytes. Nature. 1982;300(5890):368-370.
DOI 10.1038/ 300368A0

Kaczorowski D.J., Scott M.J., Pibris J.P., Afrazi A., Nakao A.,
Edmonds R.D., Kim S., Kwak J.H., Liu Y., Fan J., Billiar T.R.
Mammalian DNA is an endogenous danger signal that stimulates
local synthesis and release of complement factor B. Mol. Med.
2012;18(1):851-860. DOI 10.2119/molmed.2012.00011

Krieg A.M. CpG motifs in bacterial DNA and their immune effects.
Annual Rev. Immunol. 2002;20(1):709-760. DOI 10.1146/
annurev.immunol.20.100301.064842

Laktionov P.P., Tamkovich S.N., Rykova E.Yu., Bryzgunova O.E.,
Starikov A.V., Kuznetsova N.P., Sumarokov S.V., Kolomiets S.A.,
Sevostianova N.V., Vlassov V.V. Extracellular circulating nucleic
acids in human plasma in health and disease. Nucleosides
Nucleotides Nucleic Acids. 2004;23(6-7):879-883. DOI 10.1081/
NCN-200026035

Ledoux L. Uptake of DNA by living cells. Prog. Nucleic Acid
Res. Mol. Biol. 1965;4:231-267. DOI 10.1016/S0079-6603(08)
60790-4

Likhacheva A.S., Nikolin V.P., Popova N.A., Dubatolova T.D.,
Strunkin
D.N., Rogachev V.A., Sebeleva T.E., Erofeev I.S., Bogachev
S.S., Yakubov L.A., Shurdov M.A. Integration of human
DNA fragments into the cell genomes of certain tissues from
adult mice treated with cytostatic cyclophosphamide in combination
with human DNA. Gene Ther. Mol. Biol. 2007;11(2):
185-202

Likhacheva A.S., Rogachev V.A., Nikolin V.P., Popova N.A., Shi-lov
A.G., Sebeleva T.E., Strunkin D.N., Chernykh E.R., Gel’fgat
E.L., Bogachev S.S., Shurdov M.A. Involvement of exogenous
DNA in the molecular processes in somatic cell. Informatsionnyy
Vestnik VOGiS = The Herald of Vavilov Society for
Geneticists and Breeders. 2008;12(3):426-473 (in Russian)

Ludwig A.K., Giebel B. Exosomes: small vesicles participating in
intercellular communication. Int. J. Biochem. Cell Biol. 2012;
44(1):11-15. DOI 10.1016/j.biocel.2011.10.005

MacDougall C.A., Byun T.S., Van C., Yee M.C., Cimprich K.A.
The structural determinants of checkpoint activation. Genes
Dev. 2007;21(8):898-903. DOI 10.1101/gad.1522607

Medzhitov R., Janeway C. Innate immune recognition: mechanisms
and pathways. Immunol. Rev. 2000;173(1):89-97. DOI
10.1034/j.1600-065X.2000.917309.x

Palka-Santini M., Schwarz-Herzke B., Hösel M., Renz D.,
Auerochs
S., Brondke H., Doerfler W. The gastrointestinal tract
as the portal of entry for foreign macromolecules: fate of DNA
and proteins. Mol. Genet. Genomics. 2003;270(3):201-215. DOI
10.1007/S00438-003-0907-2

Petrova D.D., Dolgova E.V., Proskurina A.S., Ritter G.S., Ruzanova
V.S., Efremov Y.R., Potter E.A., Kirikovich S.S., Levites E.V.,
Taranov O.S., Ostanin A.A., Chernykh E.R., Kolchanov N.A.,
Bogachev S.S. The new general biological property of stem-like
tumor cells (Part II: Surface molecules, which belongs to distinctive
groups with particular functions, form a unique pattern
characteristic of a certain type of tumor stem-like cells). Int. J.
Mol. Sci. 2022;23(24):15800. DOI 10.3390/ijms232415800

Pisetsky D.S., Ullal A.J. The blood nucleome in the pathogenesis of
SLE. Autoimmun. Rev. 2010;10(1):35-37. DOI 10.1016/j.autrev.
2010.07.007

Potter E.A., Dolgova E.V., Proskurina A.S., Minkevich A.M.,
Efremov Y.R., Taranov O.S., Omigov V.V., Nikolin V.P., Popova
N.A., Bayborodin S.I., Ostanin A.A., Chernykh E.R., Kolchanov
N.A., Shurdov M.A., Bogachev S.S. A strategy to eradicate
well-developed
Krebs-2 ascites in mice. Oncotarget. 2016;
7(10):11580-11594. DOI 10.18632/oncotarget.7311

Potter E.A., Dolgova E.V., Proskurina A.S., Efremov Y.R., Minkevich
A.M., Rozanov A.S., Peltek S.E., Nikolin V.P., Popova N.A.,
Seledtsov I.A., Molodtsov V.V., Zavyalov E.L., Taranov O.S.,
Baiborodin S.I., Ostanin A.A., Chernykh E.R., Kolchanov N.A.,
Bogachev S.S. Gene expression profiling of tumor-initiating
stem cells from mouse Krebs-2 carcinoma using a novel marker
of poorly differentiated cells. Oncotarget. 2017;8(6):9425-9441.
DOI 10.18632/ONCOTARGET.14116

Potter E.A., Dolgova E.V., Proskurina A.S., Ruzanova V.S., Efremov
Y.R., Kirikovich S.S., Oshikhmina S.G., Mamaev A.L.,
Taranov
O.S., Bryukhovetskiy A.S., Grivtsova L.U., Kolchanov
N.A., Ostanin A.A., Chernykh E.R., Bogachev S.S. Stimulation
of mouse hematopoietic stem cells by angiogenin and DNA
preparations. Braz. J. Med. Biol. Res. 2024;57:e13072. DOI
10.1590/1414-431X 2024E13072

Raposo G., Stoorvogel W. Extracellular vesicles: exosomes, microvesicles,
and friends. J. Cell Biol. 2013;200(4):373-383. DOI
10.1083/JCB.201211138

Rashed M.H., Bayraktar E., Helal G.K., Abd-Ellah M.F., Amero P.,
Chavez-Reyes A., Rodriguez-Aguayo C. Exosomes: from garbage
bins to promising therapeutic targets. Int. J. Mol. Sci. 2017;
18(3): 538. DOI 10.3390/IJMS18030538

Ratajczak J., Wysoczynski M., Hayek F., Janowska-Wieczorek A.,
Ratajczak M.Z. Membrane-derived microvesicles: important
and underappreciated mediators of cell-to-cell communication.
Leukemia. 2006;20(9):1487-1495. DOI 10.1038/sj.leu.2404296

Ritter G., Dolgova E.V., Petrova D.D., Efremov Y.R., Proskurina
A.S., Potter E.A., Ruzanova V.S., Kirikovich S.S., Levites
E.V., Taranov O.S., Ostanin A.A., Chernykh E.R., Kolchanov
N.A., Bogachev S.S. The new general biological property
of stem-like tumor cells (Part I. Peculiarities of the process of
the double-stranded DNA fragments internalization into stemlike
tumor cells). Front. Genet. 2022;13:954395. DOI 10.3389/
fgene.2022.954395

Ronquist G. Prostasomes are mediators of intercellular communication:
from basic research to clinical implications. J. Intern.
Med. 2012;271(4):400-413. DOI 10.1111/j.1365-2796.2011.
02487.x

Ruzanova V., Proskurina A., Efremov Y., Kirikovich S., Ritter G.,
Levites
E., Dolgova E., Potter E., Babaeva O., Sidorov S.,
Taranov O., Ostanin A., Chernykh E., Bogachev S. Chronometric
administration of cyclophosphamide and a double-stranded
DNA-mix at interstrand crosslinks repair timing, called “Karanahan”
therapy, is highly efficient in a weakly immunogenic
Lewis carcinoma model. Pathol. Oncol. Res. 2022;28:1610180.
DOI 10.3389/PORE.2022.1610180

Sakamoto Y., Ochiya T., Yoshioka Y. Extracellular vesicles in the
breast cancer brain metastasis: physiological functions and clinical
applications. Front. Hum. Neurosci. 2023;17:1278501. DOI
10.3389/FNHUM.2023.1278501

Saukkonen K., Lakkisto P., Varpula M., Varpula T., Voipio-Pulkki
L.M., Pettilä V., Pulkki K. Association of cell-free plasma
DNA with hospital mortality and organ dysfunction in intensive
care unit patients. Intensive Care Med. 2007;33(9):1624-1627.
DOI 10.1007/s00134-007-0686-z

Schubbert R., Lettmann C., Doerfler W. Ingested foreign (phage
M13) DNA survives transiently in the gastrointestinal tract and
enters the bloodstream of mice. Mol. Gen. Genet. 1994;242(5):
495-504. DOI 10.1007/BF00285273

Schubbert R., Renz D., Schmitz B., Doerfler W. Foreign (M13) DNA
ingested by mice reaches peripheral leukocytes, spleen, and liver
via the intestinal wall mucosa and can be covalently linked to
mouse DNA. Proc. Natl. Acad. Sci. USA. 1997;94(3):961-966.
DOI 10.1073/PNAS.94.3.961

Schubbert R., Hohlweg U., Renz D., Doerfler W. On the fate of
orally ingested foreign DNA in mice: chromosomal association
and placental transmission to the fetus. Mol. Gen. Genet. 1998;
259(6):569-576. DOI 10.1007/S004380050850

Sharma S., Fitzgerald K.A. Innate immune sensing of DNA. PLoS
Pathog.
2011;7(4):e1001310. DOI 10.1371/JOURNAL.PPAT.
1001310

Sibbald S.J., Eme L., Archibald J.M., Roger A.J. Lateral gene transfer
mechanisms and pan-genomes in eukaryotes. Trends Parasitol.
2020;36(11):927-941. DOI 10.1016/J.PT.2020.07.014

Simons M., Raposo G. Exosomes – vesicular carriers for intercellular
communication. Curr. Opin. Cell Biol. 2009;21(4):575-581.
DOI 10.1016/j.ceb.2009.03.007

Soucy S.M., Huang J., Gogarten J.P. Horizontal gene transfer:
building the web of life. Nat. Rev. Genet. 2015;16(8):472-482.
DOI 10.1038/NRG3962

Takeshita F., Ishii K.J. Intracellular DNA sensors in immunity.
Curr. Opin. Immunol. 2008;20(4):383-388. DOI 10.1016/j.coi.
2008.05.009

Tetta C., Bruno S., Fonsato V., Deregibus M.C., Camussi G. The
role of microvesicles in tissue repair. Organogenesis. 2011;7(2):
105-115. DOI 10.4161/org.7.2.15782

Vatolin S.Y., Okhapkina E.V., Matveeva N.M., Shilov A.G., Baiborodin
S.I., Philimonenko V.V., Zhdanova N.S., Serov O.L.
Scheduled perturbation in DNA during in vitro differentiation
of mouse embryo-derived cells. Mol. Reprod. Dev. 1997;
47(1):1-10. DOI 10.1002/(SICI)1098-2795(199705)47:1<1::
AID-MRD1>3.0.CO;2-R

Xia J., Guo Z., Yang Z., Han H., Wang S., Xu H., Yang X., Yang F.,
Wu Q., Xie W., Zhou X., Dermauw W., Turlings T.C.J., Zhang Y.
Whitefly hijacks a plant detoxification gene that neutralizes plant
toxins. Cell. 2021;184(7):1693-1705.e17. DOI 10.1016/J.CELL.
2021.02.014

Yakubov L., Rogachev V., Likhacheva A., Bogachev S., Sebeleva
T., Shilov A., Baiborodin S., Petrova N., Mechetina L., Shurdov
M., Wickstrom E. Natural human gene correction by small
extracellular genomic DNA fragments. Cell Cycle. 2007;6(18):
2293-2301. DOI 10.4161/cc.6.18.4729

Zou L. Single- and double-stranded DNA: building a trigger of
ATR-mediated DNA damage response. Genes Dev. 2007;21(8):
879-885. DOI 10.1101/gad.1550307

